# Integrated paediatric fever management and antibiotic over-treatment in Malawi health facilities: data mining a national facility census

**DOI:** 10.1186/s12936-016-1439-7

**Published:** 2016-08-04

**Authors:** Emily White Johansson, Katarina Ekholm Selling, Humphreys Nsona, Bonnie Mappin, Peter W. Gething, Max Petzold, Stefan Swartling Peterson, Helena Hildenwall

**Affiliations:** 1 Department of Women’s and Children’s Health, International Maternal and Child Health, Uppsala University, Uppsala, Sweden; 2 Integrated Management of Childhood Illness (IMCI) Unit, Ministry of Health, Lilongwe, Malawi; 3 Spatial Ecology and Epidemiology Group, Department of Zoology, University of Oxford, Oxford, UK; 4 The Sahlgrenska Academy, Center for Applied Biostatistics, University of Gothenburg, Gothenburg, Sweden; 5 Global Health-Health Systems and Policy Research Group, Department of Public Health Sciences, Karolinska Institutet, Stockholm, Sweden; 6 School of Public Health, College of Health Sciences, Makerere University, Kampala, Uganda

**Keywords:** Antibiotic resistance, IMCI, Malaria, Diagnosis, Child health, Fever case management

## Abstract

**Background:**

There are growing concerns about irrational antibiotic prescription practices in the era of test-based malaria case management. This study assessed integrated paediatric fever management using malaria rapid diagnostic tests (RDT) and Integrated Management of Childhood Illness (IMCI) guidelines, including the relationship between RDT-negative results and antibiotic over-treatment in Malawi health facilities in 2013–2014.

**Methods:**

A Malawi national facility census included 1981 observed sick children aged 2–59 months with fever complaints. Weighted frequencies were tabulated for other complaints, assessments and prescriptions for RDT-confirmed malaria, IMCI-classified non-severe pneumonia, and clinical diarrhoea. Classification trees using model-based recursive partitioning estimated the association between RDT results and antibiotic over-treatment and learned the influence of 38 other input variables at patient-, provider- and facility-levels.

**Results:**

Among 1981 clients, 72 % were tested or referred for malaria diagnosis and 85 % with RDT-confirmed malaria were prescribed first-line anti-malarials. Twenty-eight percent with IMCI-pneumonia were not prescribed antibiotics (under-treatment) and 59 % ‘without antibiotic need’ were prescribed antibiotics (over-treatment). Few clients had respiratory rates counted to identify antibiotic need for IMCI-pneumonia (18 %). RDT-negative children had 16.8 (95 % CI 8.6–32.7) times higher antibiotic over-treatment odds compared to RDT-positive cases conditioned by cough or difficult breathing complaints.

**Conclusions:**

Integrated paediatric fever management was sub-optimal for completed assessments and antibiotic targeting despite common compliance to malaria treatment guidelines. RDT-negative results were strongly associated with antibiotic over-treatment conditioned by cough or difficult breathing complaints. A shift from malaria-focused ‘test and treat’ strategies toward ‘IMCI with testing’ is needed to improve quality fever care and rational use of both anti-malarials and antibiotics in line with recent global commitments to combat resistance.

**Electronic supplementary material:**

The online version of this article (doi:10.1186/s12936-016-1439-7) contains supplementary material, which is available to authorized users.

## Background

Since the 1990s, the World Health Organization (WHO) and United Nations Children’s Fund (UNICEF) have promoted the Integrated Management of Childhood Illness (IMCI) strategy in low- and middle-income countries to effectively manage the most common causes of child morbidity and mortality in an integrated manner [1]. It is well recognized that integrated protocols are critical for optimally managing the sick child in order to address co-morbidities and to differentiate among illnesses with overlapping symptoms [2].

Fever is a common symptom of many childhood illnesses in sub-Saharan Africa. For many years however, the IMCI strategy promoted presumptive malaria treatment of paediatric fevers in malaria-endemic African settings given high malaria mortality rates and the lack of other defining features for clinical management. While additional IMCI algorithms have been available to clinically differentiate other fever causes, the presumption of all fevers as ‘malaria’ has often impeded probing for these other conditions [3].

In 2010, WHO revised malaria treatment guidelines to recommend diagnosis of all suspected malaria cases prior to treatment given the increasing availability of malaria rapid diagnostic tests (RDT) [4]. This policy shift has great potential to improve rational drug use and quality fever care [5], although studies indicate common inappropriate treatment of RDT-negative patients with anti-malarial or antibiotic drugs [6]. These findings suggest poor integration of RDT into the IMCI framework, although few studies have explicitly examined integrated paediatric fever management and available evidence is largely derived from limited hospital settings [7–12]. There is also limited understanding of factors associated with non-adherence to clinical guidelines, notably antibiotic over-treatment, which is a particular concern in the era of test-based malaria case management [13]. This concern reflects studies showing widespread antibiotic prescriptions for test-negative cases and not according to established clinical guidelines [1, 6, 8].

Malawi recently adopted the ‘test and treat’ strategy in its National Malaria Strategic Plan and began nationwide RDT deployment in July 2011 [14]. A national facility study conducted prior to RDT implementation showed low availability of functional microscopy for malaria diagnosis [15], and common non-compliance to negative blood smear results [16]. Similar evidence is needed from the post-RDT implementation period in Malawi with an expanded analysis of how RDT and IMCI are used together during outpatient consultations.

In this paper, a national facility census conducted in Malawi in 2013–2014 was analyzed to examine integrated paediatric fever management using RDT and IMCI, including other presenting complaints, completed assessments, diagnoses/classifications, and treatment prescriptions [17]. The large number of facilities audited coupled with the broad data collection scope provides a unique opportunity to investigate the association between RDT results and antibiotic over-treatment. Classification trees are well suited for such an analysis since numerous influences on different levels may shape the complex nature of the clinical encounter and there may be complicated inter-relationships among variables that are not well defined in advance or easily detected using standard statistical methods [18]. Indeed, traditional regression models assume a uniform influence of the exposure on an outcome unless an interaction is specified, which is unrealistic in real-life contexts and complicates results interpretation. It is also a challenging situation to model if numerous variables may influence an examined relationship and there is limited a priori knowledge of these potentially complex interactions in order to define a clear hypothesis for statistical testing.

## Methods

### Study setting

Malaria is endemic in most parts of Malawi with peak transmission in November–April, although transmission has declined in recent years. Malawi’s health system is primarily comprised of government-run facilities and publicly supported facilities run by the Christian Health Association of Malawi (CHAM) [19]. This system contains three main tiers: regional hospitals, district hospitals and health centres. The primary tier is the health centre, which provides essential services, including family planning, antenatal care and other outpatient services. The secondary tier is the district hospital, which are referral facilities that also provide in-patient care, laboratory diagnostics and maternity care. The tertiary level is the central or regional hospital, which are teaching and research hospitals that provide specialized medical care. Community-based sick child services are also provided in Malawi but are not included in this facility-based assessment.

### Survey methods

The Malawi Service Provision Assessment (SPA) was conducted in June 2013–February 2014 by the Ministry of Health and the Demographic and Health Survey (DHS) programme, which includes facility and laboratory audits, observed consultations, patient exit interviews, and health worker interviews. Survey methods are described elsewhere [17]. Briefly, Malawi SPA 2013–2014 was designed as a census of all formal public and private facilities in the country. At each facility, clients attending the facility on the interview date were systematically selected for observation. The expected patient load for outpatient sick child curative services on that date was estimated in advance and every Nth client attending the facility on that date was selected for observation in order to yield no more than 15 observations per facility. Clients were eligible to participate if they were under 5 years of age and presented with an illness complaint and not an exclusive injury or non-disease condition. Sick child observations aim to assess clinical practices according to Malawi IMCI guidelines [20]. During the exit interview, a limited re-examination protocol was conducted by clinicians, nurses or nurse midwives specifically trained to take a 60-s respiratory rate count and temperature reading by thermometer.

Ethical approval for collection of these data was obtained by the DHS programme from the Department of Health and Human Services Institutional Review Board (IRB) and the host country IRB, which includes authorization to distribute unrestricted survey files for secondary analysis purposes upon receipt of a research proposal. Written informed consent was obtained separately from health workers and caregivers prior to participation in the observation, exit interview and re-examination [17].

### Inclusion criteria

**Fig. 1:**
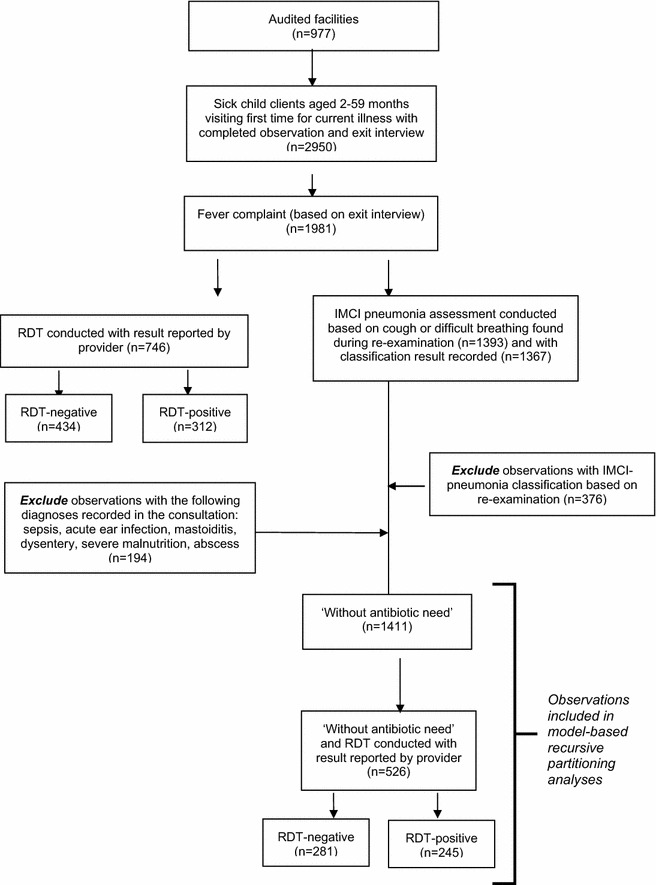
Study sample

Children aged 2 months–5 years attending an observed outpatient consultation as a first-time visit for an illness were included if they had a fever complaint and provided consent for the observed consultation and exit interview (Fig. 1). The antibiotic over-treatment analysis applied only to those clients ‘without antibiotic need’ as defined below.

### Integrated paediatric fever management

Table 1 defines key measures of integrated paediatric fever management reported in this paper, including other complaints, completed assessments, classifications/diagnoses and drug prescriptions for RDT-confirmed malaria, IMCI-pneumonia, and clinical diarrhoea.

**Table 1:** Description of integrated paediatric fever management variables

Description	
*Main complaints or danger signs*	
Fever	During the exit interview, the caregiver is separately asked about each of the main symptoms or danger signs listed here
Cough or difficult breathing (CDB)	
Watery or frequent stools	
Danger signs (any below):	
Lethargy or excessive sleepiness	
Vomits everything	
Convulsions	
Inability to drink, eat or breastfeed	
Ear problem	During the exit interview, the caregiver is subsequently asked about other reasons for bringing the child to this facility today and the response categories are listed here
Eye problem	
Skin problem	
Other issue	
*Assessments*	
Asked about or mentioned (insert complaint)	During the consultation, the interviewer silently records the performance of physical examinations. Those listed here are general assessments for presenting complaints of fever, cough or difficult breathing or diarrhea. Assessments reported in this paper are those based on Malawi IMCI algorithms unless otherwise noted^a^
Took the child’s temperature or felt body for hotness	
Counter respiration (breaths) for 60 s	
Checked skin turgor for dehydration	
Checked pallor by looking at palms^a^	
Looked into the child’s mouth^a^	
Checked for neck stiffness	
Undressed child (up to shoulders/down to ankles)^a^	
*Classifications/diagnoses*	
RDT-confirmed malaria	After the consultation, the provider is asked if a malaria RDT was conducted anywhere in the facility prior to coming into the consultation room that day and if so, the provider is asked to report the test result if seen
IMCI-classified non-severe pneumonia	During the exit interview, there is a limited re-examination conducted by a trained provider that includes a 60-s respiratory rate count if cough or difficult breathing is present. IMCI-pneumonia classification (non-severe) is defined as reported cough or difficult breathing and a respiratory rate of 50 breaths or more per minute (2 up to 12 months) or 40 breaths or more per minute (12 months up to 5 years)
Clinical diarrhoea	During the consultation, the following recorded diagnoses for diarrhea or dehydration are included in this definition: diarrhoea, dysentery, amoebiasis, other digestive/intestinal issue, mild dehydration, moderate dehydration or severe dehydration
*Treatment prescriptions*	
Anti-malarial prescriptions	After the consultation, the provider is asked to report treatments prescribed to the client and a hierarchical coding was used to assign the more appropriate prescription to the observation. First-line anti-malarial prescription is defined as artemether/artesunate (oral, injection or suppository) or ACT/AL (oral). Second-line is quinine (oral or injection), amodiaquine (oral), fansidar (oral) or other anti-malarial (oral or injection). Anti-malarial over-treatment is any anti-malarial prescription for an RDT-negative result
Antibiotic prescriptions	After the consultation, the provider is asked to report treatments prescribed to the client and a hierarchical coding was used to assign the more appropriate prescription to the observation. First-line antibiotic prescription is defined as benzyl penicillin injection or amoxicillin (capsule or syrup). Second-line is cotrimoxazole (syrup or tablet) or other antibiotic (injection, syrup or capsule). Antibiotic over-treatment is the main outcome and is defined in the text
ORS and zinc prescriptions	After the consultation, the provider is asked to report treatments prescribed to the client. ORS and zinc is defined as a prescription of zinc and [home ORT (plan A) or initial ORT in facility (plan B) or intravenous fluids (plan C)]

^a^ Checked for palm pallor, looked into child’s mouth and undressed child to examine (up to shoulders/down to ankles) are general fever assessments for rash, petechiae due to meningitis or other febrile causes

### Antibiotic over-treatment

Antibiotic over-treatment or any antibiotic prescription ‘without antibiotic need’ is defined as a IMCI-pneumonia negative classification based on re-examination and additionally excluding the following diagnoses recorded during the consultation: sepsis, acute ear infection, mastoiditis, dysentery, abscess, or severe malnutrition. Any antibiotic prescription includes any antibiotic injection (benzyl penicillin or other) or antibiotic capsule, syrup or tablet (amoxicillin, cotrimoxazole or other) that the provider reported was prescribed during the consultation. Table 1 defines the main predictors: RDT conducted (yes or no) and RDT result (positive or negative). Table 2 defines the other 38 input variables at different levels in the analysis, which includes malaria risk (infection prevalence) values for 2013–2014 linked to datasets through geocoded facility locations, and transmission season estimates derived from facility locations and interview dates [21, 22].

**Table 2:** Description of input variables in the antibiotic over-treatment analysis

Input	Description	Source
Main		
RDT done	RDT done prior to consultation (yes or no)	Provider interview
RDT result	RDT result (positive or negative)	Provider interview
Patient		
Caregiver sex	Gender (male or female)	Exit interview
Child sex	Gender (male or female)	Observation
Caregiver age	Age (numeric: 11–74 years)	Exit interview
Diarrhoea	Diarrhea complaint (yes or no)	Exit interview
CDB	Cough or difficult breathing (yes or no)	Exit interview
Danger sign	Any danger sign complaint (yes or no)	Exit interview
Temperature	Temperature (numeric: 35°–40.8°)	Re-examination
Illness duration	Illness duration (numeric: 0–60 days)	Exit interview
Nearest facility	Nearest facility to home (yes or no)	Exit interview
Clinical examination	Counted breaths for 60 s (yes or no)	Observation
Consultation length	Derived from consultation start and end times (numeric: 0–307 min)	Observation
Consultation start hour	Derived from consultation start time (numeric: 7:00–17:00)	Observation
Wait time	Reported wait from arrival to consultation (numeric: 0–600 min)	Exit interview
Provider		
Provider sex	Gender (male or female)	Observation
Job qualification	Doctor/clinical officer/technician or medical assistant or nurse/midwife/HSA	Observation
Supervisor status	Supervisor or in-charge (yes or no)	Provider interview
Experience	Year received current job qualification (numeric: 1950–2014)	Provider interview
Work hours	Average work hours per week (numeric: 1–90 h per week)	Provider interview
Training	RDT training (ever received or not)	Provider interview
Training	IMCI training (ever received or not)	Provider interview
Supervision	Provider supervision (ever received or not)	Provider interview
Supervision quality	Discussed work issues during most recent supervisory visit (yes or no)	Provider interview
Facility		
Malaria risk	P*f*PR in 2–10 year olds (numeric: 0.0–0.4)	Malaria Atlas Project
Transmission season	Transmission season (peak or off-peak)	MARA
Location	Residence (urban or rural)	Facility audit
Region	Region (central or north or south)	Facility audit
Facility type	Hospital (central, district, rural, other) or other facility (centre, post, dispensary, clinic)	Facility audit
Managing authority	Government or CHAM/other	Facility audit
Management	Routine management meetings (yes or no)	Facility audit
Staffing	Total staff doctors (numeric: 0–119)	Facility audit
External supervision	External supervisory visit to facility (ever received or not)	Facility audit
User fees	Routine general user fees (yes or no)	Facility audit
Medicine stocks	Antibiotic (any type available or not)	Facility audit
Medicine stocks	Anti-malarial (any type available or not)	Facility audit
Supply stocks	RDT (observed valid or not in either service area or laboratory)	Facility audit
Supply stocks	Facility or staff timer (available or not)	Facility audit
Guidelines	RDT job aid or guidelines (available or not)	Facility audit
Guidelines	IMCI guidelines (available or not)	Facility audit

### Data analyses

Visual content mapping depicted the potential inter-relationships of input variables on clinical treatment decisions using the Visual Understanding Environment 3.3.0 (Tufts University, Somerville, MA, USA) [23]. Frequencies and cross-tabulations were calculated using weights to account for the unequal probabilities of selection due to differing client volumes at facilities on the interview date. Standard error estimation accounted for clustering of client observations within facilities. The level of statistical significance was set to 0.05. Stata 13.1 (Stata Corp., College Station, TX, USA) was used for analyses.

Classification trees were used to learn the relative importance of main predictors (RDT conducted and RDT result) and their inter-relationships with other input variables on the binary outcome of antibiotic over-treatment. A model-based recursive partitioning approach [24] was used in this analysis that embeds a parametric model into a recursive partitioning algorithm in order to identify sub-groups within the dataset where there may be different patterns of association between the main predictor and outcome. The model is subsequently re-fit to identified sub-groups, known as nodes, in order to describe different and complex relationships among variables with respect to an outcome across these sub-groups.

In this analysis, a mixed-effects logistic regression model was initially fit to estimate the relationship between the RDT result (or RDT conducted) and antibiotic over-treatment, with observations nested within facility identifiers. The potential influence of 38 other variables on this relationship was learned through recursive partitioning that allowed for detection of sub-group interactions and estimation of random effects parameters [25]. Parameter instability was repeatedly assessed over the set of 38 potential partitioning variables using a Bonferroni-corrected significance level of 0.05. Nodes were split according to the variable, resulting in highest instability, known as a significant classifier. This process was repeated for each resulting sub-group until the minimal node size of 20 observations was reached or no additional significant classifiers were identified. This approach yields a tree fitted to models associated with each terminal node along with estimated odds ratios or other coefficients for the effect of the main predictor on an outcome in each resulting sub-group. R version 3.2.2 and the ‘partykit’ package was used for this analysis [26, 27].

## Results

The Malawi Service Provision Assessment 2013–2014 included 977 facilities out of 1060 on the Ministry of Health master facility list with non-response due to refusal (3 %), closure (2 %), inaccessibility (2 %), or other issue (1 %). A total of 2950 sick child clients met inclusion criteria and 1981 reported fever complaints (Fig. 1). Additional files 1, 2 describe characteristics of febrile clients with RDT results and receiving antibiotic over-treatment respectively.

### Complaints

**Fig. 2:**
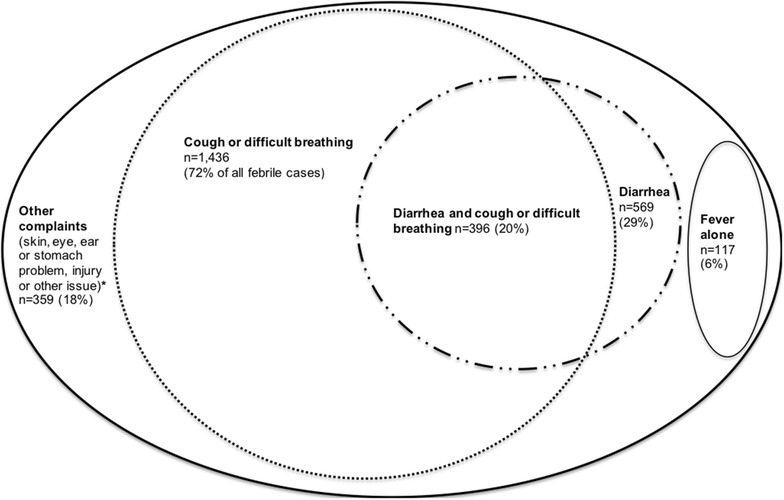
Other complaints among clients with fever complaints, Malawi health facilities, 2013–2014. Totals may not sum to 1981 cases due to multiple reported symptoms. Any danger sign was reported in 1021 (52 %) of these observations. Symptom complaints are based on caregiver reports during exit interviews. Fever alone is without any other reported complaint or danger sign

Among 1981 eligible clients, 1436 (72 %) also reported cough or difficult breathing (CDB) complaints; 569 (29 %) had diarrhoea complaints; 359 (18 %) reported other complaints including skin problems, eye problems, ear problems, stomach problems, injuries or other issues; 1021 (52 %) reported any danger sign (lethargy, inability to drink or breastfeed, convulsions or vomits everything); 117 (6 %) reported fever alone with no other complaint or danger sign (Fig. 2).

### Assessments

Among 1981 eligible clients, 1684 (85.0 %) either spontaneously mentioned the fever complaint or were asked about fever by the provider during the consultation; 1386 (70.0 %) had their temperature taken or body felt for hotness; 1426 (72.0 %) had a malaria RDT done prior to the consultation or were referred for malaria diagnosis; 44 (2.2 %) had neck checked for stiffness; 524 (26.5 %) had palm pallor checked; 185 (9.3 %) had the inside of their mouth checked; and 563 (28.4 %) were undressed for examination (up to shoulders/down to ankles). Among 1436 clients with both fever and CDB complaints, 256 (17.8 %) had respiratory rates counted for 60 s. Among 569 clients with fever and diarrhoea complaints, 98 (17.3 %) had skin turgor checked for dehydration (Table 3).

**Table 3:** Assessments of clients with fever complaints, Malawi health facilities, 2013–2014

	N Assessed	% Assessed (95% CI)
*Fever complaint*	*1981*	
Fever mentioned or asked about by provider	1684	85.0 (82.8–87.2)
Temperature taken or body felt for hotness	1386	70.0 (65.5–74.1)
RDT done prior to consultation or referral for malaria diagnosis^a^	1426	72.0 (69.0–74.7)
Checked neck for stiffness	44	2.2 (1.4–3.5)
Checked for pallor by looking at palms	524	26.5 (23.5–29.6)
Looked into child’s mouth	185	9.3 (7.4–11.6)
Undressed child to examine (up to shoulders/down to ankles)	563	28.4 (25.2–31.9)
*Fever and CDB complaint*	*1436*	
Both symptoms mentioned or asked about by provider	1010	70.3 (66.7–73.7)
Counted breaths for 60 s	256	17.8 (14.8–21.2)
*Fever and diarrhea complaint*	*569*	
Both symptoms mentioned or asked about by provider	307	53.9 (48.3–59.4)
Checked skin turgor for dehydration	98	17.3 (13.3–22.1)

Symptom complaints are based on caregiver reports during exit interviews. Completed assessments are based on recorded observations during consultations

^a^RDT done prior to consultation is based on provider reports that RDT was done prior to the consultation. Referral for malaria diagnosis is based on caregiver reports during the exit interview that the provider who treated the child instructed him/her to take the child to see another provider, or to go to the laboratory in this facility for a finger or heel stick for blood to be taken for testing

### Anti-malarial prescriptions

Among 1981 eligible clients, 746 (37.7 %) had malaria RDT conducted prior to the consultation with results reported. Among 312 with reported RDT-positive results, 265 (85.1 %) received first-line anti-malarial prescriptions; 22 (7.0 %) received second-line anti-malarial prescriptions; and 25 (7.9 %) received no anti-malarial prescription (anti-malarial under-treatment). Among 434 with reported RDT-negative results, 44 (10.2 %) received any anti-malarial prescription (anti-malarial over-treatment) (Table 4).

**Table 4:** Anti-malarial and antibiotic prescriptions for clients with fever complaints, Malawi health facilities, 2013–2014

	N	% prescribed treatment (95% CI)
*Fever complaint*	*1981*	
*RDT done prior to consultation or referral for malaria diagnosis*	*1426*	
*RDT done prior to consultation with result reported*	*746*	
*RDT-positive result*	*312*	
First-line anti-malarial prescription	265	85.1 (77.5–90.4)
Second-line anti-malarial prescription	22	7.0 (4.4–10.8)
No anti-malarial prescription	25	7.9 (3.6–16.7)
*RDT-negative result*	*434*	
Any anti-malarial prescription (over-treatment)	44	10.2 (6.8–14.9)
*IMCI pneumonia assessment with result reported*	*1367*	
*IMCI pneumonia (non-severe) positive classification*	*376*	
First-line antibiotic prescription	148	39.4 (32.3–46.9)
Second-line antibiotic prescription	123	32.7 (26.3–39.8)
No antibiotic prescription	105	27.9 (20.7–36.5)
*‘Without antibiotic need’*	*1411*	
Any antibiotic prescription (over-treatment)	830	58.8 (55.1–62.4)

Table 1 defines assessments and treatments reported in the above table. Anti-malarial under-treatment is defined as no anti-malarial prescription for an RDT-positive result. Anti-malarial over-treatment is defined as any anti-malarial prescription for an RDT-negative result. Antibiotic under-treatment is defined as no antibiotic prescription for a positive IMCI pneumonia classification. Antibiotic over-treatment is defined as any antibiotic prescription ‘without antibiotic need’, which excludes clients with IMCI-pneumonia based on re-examination and additionally excludes clients given the following diagnoses during the consultation: sepsis, acute ear infection, mastoiditis, dysentery, abscess, or severe malnutrition

### Antibiotic prescriptions

Among 1981 eligible clients, 1367 (70.3 %) were assessed for IMCI pneumonia with results reported. Among 376 with non-severe IMCI-pneumonia from re-examination, 148 (39.4 %) received first-line antibiotic prescriptions; 123 (32.7 %) received second-line antibiotic prescriptions; and 105 (27.9 %) received no antibiotic prescription (antibiotic under-treatment). There were 917 with a negative IMCI-pneumonia classification and a total of 1411 were further categorized as ‘without antibiotic need’. Among 1411 clients ‘without antibiotic need’, 830 (58.8 %) received any antibiotic prescription (antibiotic over-treatment) (Table 4).

### Oral rehydration solution and zinc prescriptions

Among 1981 eligible clients, 260 (13.1 %) were given diagnoses of dehydration or intestinal/digestive issue. Among 260 with these diagnoses, 187 (72.1 %) received oral rehydration solution (ORS) and 148 (56.9 %) received both ORS and zinc prescriptions.

### Antibiotic over-treatment

**Fig. 3:**
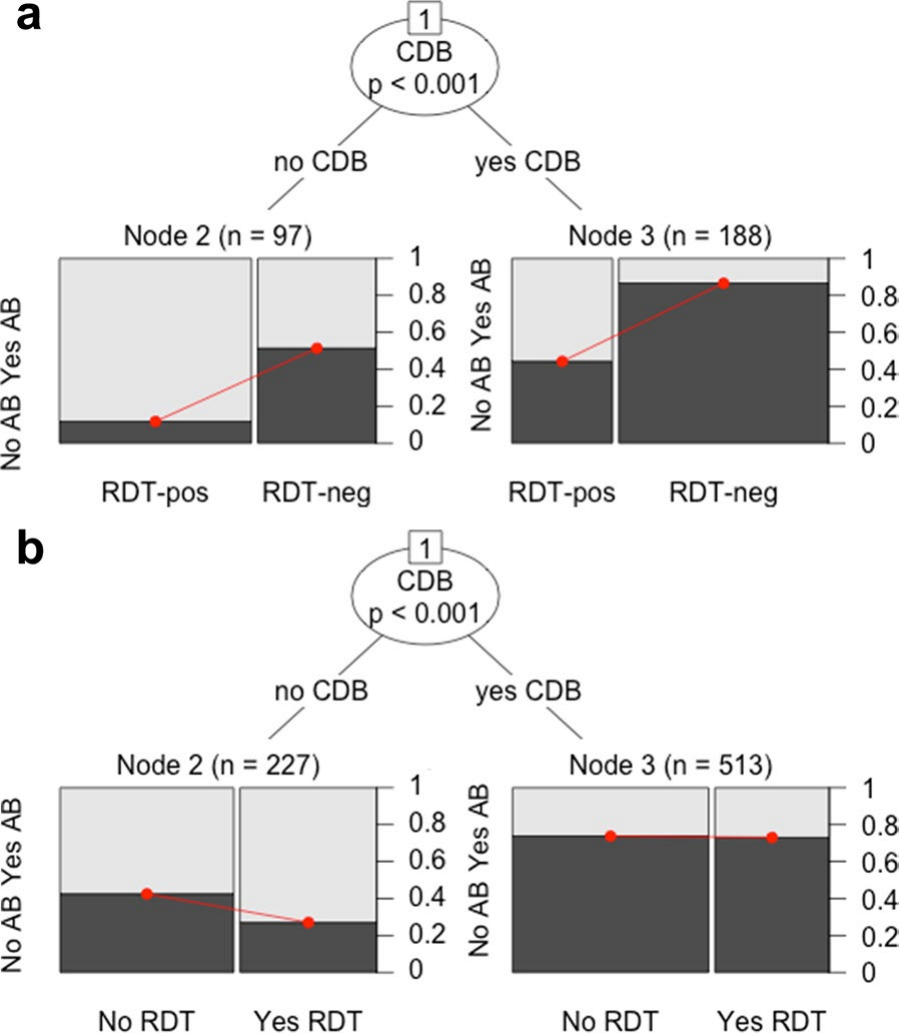
Inter-relationship between RDT result (**a**) or RDT done (**b**) and other input variables on antibiotic over-treatment, Malawi health facilities, 2013–2014. CDB refers to cough or difficult breathing complaint. AB refers to antibiotic. Table 2 lists all input variables included in the model-based recursive partitioning analysis

Among the sub-set of 526 clients ‘without antibiotic need’ and reported RDT results, RDT-negative clients had 16.8 (95 % CI 8.6–32.7) times higher antibiotic over-treatment odds compared to RDT-positive clients in the crude mixed-effects logistic regression model. CDB complaint was a statistically significant classifier of this relationship learned through recursive partitioning (p < 0.001). Figure 3a depicts all observations ‘without antibiotic need’ and the dark grey bars indicate those receiving any antibiotic prescription, or antibiotic over-treatment. This figure indicates that the split by CDB complaint is largely driven by a difference in the underlying risk of antibiotic over-treatment across groups rather than changing patterns of association between RDT results and antibiotic over-treatment. The lowest risk of antibiotic over-treatment was found among clients without CDB complaint and a positive RDT result. This risk significantly increased with the negative RDT result (Node 2: OR: 8.9; n = 97). In contrast, clients with CDB complaint already had relatively high underlying risk of antibiotic over-treatment irrespective of the RDT result and this risk similarly increased with a negative result (Node 3: OR: 5.6, n = 188). Indeed, the highest risk of antibiotic over-treatment was among clients with CDB complaint and a negative RDT result. In this group, 82 % of clients were inappropriately prescribed antibiotics according to the study definition.

Figure 3b depicts the relationship between RDT conducted prior to consultation and antibiotic over-treatment among the subset of 1411 clients ‘without antibiotic need’ either tested or not for malaria. Antibiotic over-treatment odds were reduced among clients tested compared to untested in the crude mixed-effects logistic regression model (OR: 0.48, 95 % CI 0.35–0.64). CDB complaint was a statistically significant classifier in this analysis, and testing was differently associated with the outcome if this complaint was reported (node 2 OR: 0.5, n = 227; node 3: OR: 1.0, n = 513). Conducting RDT prior to the consultation reduced antibiotic over-treatment odds among clients without CDB complaints compared to those untested, but this effect was negligible among those with this complaint.

## Discussion

Integrated paediatric fever management was sub-optimal in terms of fever assessments completed and poor antibiotic targeting, although findings suggest common compliance to malaria treatment guidelines. The RDT negative result was strongly associated with antibiotic over-treatment conditioned by CDB complaints.

In this study, only 6 % of clients with fever complaints had no other complaint or danger sign underscoring the critical need for integrated protocols to manage sick children [1–3]. It was further shown that most clients with fever complaints received a malaria test or were referred for diagnosis, and RDT-guided malaria treatment seemed common according to provider reports. This finding suggests compliance with new malaria treatment guidelines in Malawi in 2013–2014 and contrasts with previous studies showing poor adherence to negative blood smear readings prior to nationwide RDT deployment [16]. This result should be viewed in light of data limitations described later in this section and additional studies are needed to corroborate this finding.

Yet general fever assessments were less commonly conducted despite being essential for differential diagnosis. This finding is consistent with other research showing poor IMCI implementation in Malawi and other settings [28, 29]. There was also poor antibiotic targeting with both under- and over-treatment that is in part due to poorly assessing clients to identify antibiotic need. Poor antibiotic targeting in low-income settings has been documented in other research [7–12]. This study, however, is the first to our knowledge to provide large-scale evidence of integrated paediatric fever management using RDT and IMCI during outpatient consultations, including the important relationship between RDT results and antibiotic over-treatment.

These findings demonstrate the strong influence of RDT-negative results on antibiotic over-treatment and an inter-relationship with CDB complaints, which reinforces the primary importance of patient symptoms and diagnostic test results on clinical treatment decisions. This is consistent with research showing widespread antibiotic prescriptions for RDT-negative cases [8–11], and cough complaint as a main predictor of incorrect malaria treatment in Malawi [16]. The relatively small sample size may have limited detection of other inter-relationships, notably over-treatment previously documented among urban clients [30]. Nevertheless, data mining tasks are well suited to discover inter-relationships among variables with respect to an outcome, particularly if there is limited a priori knowledge of these associations [18]. These methods have been widely used in business and biomedical research but their application in global health research has been limited and should be increasingly considered where appropriate [31–35].

Taken together, these results underscore growing concerns about irrational antibiotic prescription practices in the era of test-based malaria case management [13], particularly given recent research showing viral disease is a far more common cause of paediatric fevers in various African settings compared to bacterial or parasitic infections [36, 37]. A recent World Health Assembly resolution urges countries to develop action plans to combat antibiotic resistance in the coming years [38]. A main focus for low-income countries will be to extend the reach of health systems to expand access to life-saving medicines while simultaneously strengthening quality care at facilities that could in turn improve antibiotic targeting [39]. These results highlight the need to implement IMCI and RDT together to strengthen integrated paediatric fever management and rational use of both anti-malarial and antibiotic medicines.

To this end, the IMCI algorithm has been adapted to reflect test-based malaria treatment guidance, and there are efforts to further strengthen these guidelines based on recent etiology studies [36, 37], and in recognition of its poor implementation to date [29]. However, this new IMCI adaptation lacks clarity on antibiotic indications in the fever algorithm that could in turn inadvertently promote antibiotic over-treatment [40], which has been demonstrated in other recent research [9]. It is critical that IMCI guidelines clearly indicate when antibiotics are (or are not) recommended for sick children, particularly for RDT-negative cases, and additional review of these guidelines from this perspective may be needed.

These results should be viewed in light of data limitations. First, client selection is based on sick child attendance on the interview date and do not represent clients visiting facilities on different dates/seasons, nor all sick children in Malawi. Second, providers may perform better during observations than in routine conditions biasing results towards better practices, including RDT compliance [41]. Third, assessments recorded do not include all IMCI fever assessments, notably asking about fever duration or measles history. There is also no recording of assessment quality or clinical findings. Fourth, it may be difficult for observers to recognize that certain assessments were conducted, such as checking for neck stiffness, which could underestimate results. Fifth, the re-examination was a limited protocol that only assessed the sick child for a raised respiratory rate, signs of anaemia and fever presence based on a thermometer reading. Other assessments that could potentially indicate pneumonia or antibiotic need were not assessed in the re-examination, such as chest indrawing or hypoxia.

Measurement limitations for main predictors and the outcome should also be highlighted. First, RDT results are based on provider reports without supporting documentation. The provider reports RDT results after providing information on diagnoses and prescriptions. Some providers may misreport a negative result as positive if anti-malarial medicines were prescribed to seem in compliance with guidelines. Misclassification of the positive result as negative seems less likely in this scenario. This could potentially explain common RDT compliance found in this assessment and these results should be corroborated by additional studies. Second, RDT compliance estimates are only for clients diagnosed by the consultation time and do not include blood smear or RDT results not available by the initial consultation. Facilities conducting RDT prior to the consultation may be systematically different from other facilities in ways that influence compliance, such as larger facilities with more staff and better quality care. Third, antibiotic over-treatment is notoriously difficult to measure in settings without diagnostics to differentiate bacterial from other pathogenic causes. This paper defines ‘need’ according to IMCI antibiotic indications for pneumonia and provider reported diagnostic categories requiring antibiotics: sepsis, dysentery, mastoiditis, acute ear infection, abscess, or severe malnutrition. Urinary tract infection is not a diagnostic category and is not included in this definition. Clients assigned these diagnoses may not have the underlying condition and may not need antibiotics. The ‘without antibiotic need’ definition in this study therefore underestimates true lack of need. Fourth, IMCI pneumonia can be difficult to assess even by a trained provider leading to some misclassification in either direction [42].

## Conclusion

Based on 977 facilities and 1981 eligible clients, study findings demonstrate sub-optimal integrated paediatric fever management practices in Malawi health facilities in 2013–2014. While malaria-specific assessments and RDT-guided treatment seemed common, other fever assessments were not often completed and poor antibiotic targeting was demonstrated. RDT-negative results were strongly associated with antibiotic over-treatment conditioned by CDB complaints. These results suggest moving beyond malaria-focused ‘test and treat’ strategies toward ‘IMCI with testing’ to improve quality fever care and rational use of both anti-malarial and antibiotic medicines. Integrated paediatric fever management using RDT and IMCI together is critical to improve antibiotic targeting in line with recent commitments to combat antibiotic resistance, and should be considered for inclusion in national action plans developed by malaria-endemic African countries in the next year.

## Additional files

10.1186/s12936-016-1439-7 Background characteristics of clients with fever complaints and reported RDT results, Malawi health facilities, 2013–2014.
10.1186/s12936-016-1439-7 Background characteristics of clients with fever complaints and antibiotic over-treatment, Malawi health facilities, 2013–2014.

## Abbreviations

AB: antibiotic
CDB: cough or difficult breathing
CHAM: Christian Health Association of Malawi
DHS: Demographic and Health Survey
IMCI: Integrated Management of Childhood Illness
IRB: Institutional Review Board
ORS: oral rehydration solution
RDT: malaria rapid diagnostic test
SPA: Service Provision Assessment
