# Supplementary material for: Integrated paediatric fever management and antibiotic over-treatment in Malawi health facilities: data mining a national facility census
Source: Malar J. 2016 Aug 4;15:396. doi: 10.1186/s12936-016-1439-7 (PMC4972956; doi:10.1186/s12936-016-1439-7)
Supplement: Supplementary file 1 — 10.1186/s12936-016-1439-7 Background characteristics of clients with fever complaints and reported RDT results, Malawi health facilities, 2013–2014. [file 12936_2016_1439_MOESM1_ESM.docx]

**Additional File 1: Background characteristics of clients with fever complaints and reported RDT results, Malawi health facilities, 2013-2014**

|  |  |  | **Fever complaint** | **RDT done prior to consultation** | | | | **RDT-positive result** | | | | **RDT-negative result** | | | |
| --- | --- | --- | --- | --- | --- | --- | --- | --- | --- | --- | --- | --- | --- | --- | --- |
|  |  |  | N | N | % | 95% CI | | N | % | 95% CI | | N | % | 95% CI | |
|  |  | **Total** | **1,981** | **761** | **38.4** | **34.0** | **43.1** | **312** | **41.0** | **36**.**7** | **45**.**3** | **434** | **57**.**0** | **52**.**5** | **61**.**3** |
| **Facility** | Type | Hospital (central, district, rural, other) | 705 | 232 | 32**.**9 | 23**.**7 | 43**.**7 | 82 | 35**.**5 | 27.1 | 44.9 | 143 | 61.5 | 51.2 | 70.9 |
|  |  | Health center, dispensary, clinic, post | 1,277 | 530 | 41**.**5 | 37**.**4 | 45**.**6 | 230 | 43**.**3 | 38.7 | 48.1 | 291 | 55.0 | 50.2 | 59.6 |
|  | Managing authority | Government | 1,503 | 570 | 37**.**9 | 32**.**5 | 43**.**7 | 243 | 42**.**6 | 37.6 | 47.7 | 315 | 55.3 | 50.0 | 60.5 |
|  |  | CHAM or other | 478 | 191 | 40**.**0 | 33**.**5 | 46**.**9 | 69 | 36**.**1 | 28.5 | 44.4 | 118 | 61.9 | 53.5 | 69.6 |
|  | Region | Northern | 318 | 135 | 42**.**6 | 32**.**7 | 53**.**1 | 56 | 41**.**3 | 32.3 | 50.9 | 76 | 56.4 | 47.0 | 65.3 |
|  |  | Central | 947 | 326 | 34**.**5 | 27**.**6 | 42**.**1 | 144 | 44**.**0 | 37.8 | 50.4 | 171 | 52.3 | 45.8 | 58.9 |
|  |  | Southern | 717 | 300 | 41**.**8 | 35**.**4 | 48**.**5 | 112 | 37**.**5 | 30.7 | 44.9 | 187 | 62.2 | 54.8 | 69.1 |
|  | Location | Urban | 611 | 182 | 29**.**8 | 20**.**1 | 41**.**7 | 61 | 33**.**7 | 24.9 | 43.9 | 114 | 62.3 | 50.8 | 72.6 |
|  |  | Rural | 1,370 | 579 | 42**.**3 | 38**.**2 | 46**.**5 | 251 | 43**.**2 | 38.5 | 48.1 | 320 | 55.3 | 50.4 | 60.0 |
|  | RDT stocks | RDT (at least 1 observed, valid) | 1,857 | 711 | 38**.**3 | 33**.**9 | 42**.**9 | 290 | 40**.**8 | 36.4 | 45.4 | 407 | 57.2 | 52.5 | 61.8 |
|  |  | RDT not seen, valid, available | 65 | 18 | 27**.**8 | 13**.**8 | 48**.**2 | 8 | 43**.**8 | 23.5 | 66.4 | 10 | 56.2 | 33.6 | 76.5 |
|  | Malaria risk | PfPR 2-10 years [Mean, SD] | 1,981 | 761 | [15**.**4, 7**.**2] | | | 312 | [16.3, 7.2] | | | 434 | [14.9, 7.3] | | |
|  | Transmission season | Off-peak season | 1,684 | 635 | 37**.**7 | 32**.**8 | 42**.**9 | 263 | 41**.**4 | 36.8 | 46.3 | 357 | 56.2 | 51.2 | 61.1 |
|  |  | Peak season | 298 | 126 | 42**.**4 | 33**.**5 | 51**.**8 | 49 | 38.5 | 29.5 | 48.3 | 77 | 60.8 | 50.9 | 69.8 |
| **Patient** | Child's age | Age (months) [Mean, SD] | 1,981 | 761 | [23**.**3, 15**.**4] | | | 312 | [26.4, 15.7] | | | 434 | [21.3, 14.8] | | |
|  | Maternal education | Primary school attendance | 1,303 | 495 | 37**.**9 | 32**.**8 | 43**.**4 | 216 | 43.7 | 38.9 | 48.6 | 265 | 53.6 | 48.5 | 58.6 |
|  |  | Secondary or more | 426 | 147 | 34**.**4 | 28**.**2 | 41**.**3 | 45 | 31.0 | 23.6 | 39.6 | 99 | 67.7 | 59.1 | 75.2 |
|  | CDB complaint | CDB complaint | 1,436 | 522 | 36**.**4 | 31**.**7 | 41**.**3 | 197 | 37.8 | 32.9 | 43.0 | 313 | 59.9 | 54.4 | 65.2 |
|  |  | No CDB | 545 | 239 | 43**.**9 | 37**.**2 | 50**.**8 | 115 | 47.9 | 41.3 | 54.5 | 121 | 50.5 | 43.8 | 57.3 |
|  | Diarrhea complaint | Diarrhea complaint | 569 | 213 | 37**.**3 | 31**.**4 | 43**.**7 | 86 | 40.3 | 33.4 | 47.6 | 123 | 57.6 | 50.4 | 64.5 |
|  |  | No diarrhea | 1,412 | 549 | 38**.**9 | 34**.**2 | 43**.**7 | 226 | 41.2 | 36.1 | 46.5 | 311 | 56.7 | 51.0 | 62.2 |
| **Provider** | Qualification | Doctor or clinical officer | 236 | 89 | 37**.**5 | 26**.**7 | 49**.**7 | 27 | 30.2 | 6.9 | 47.9 | 60 | 68.2 | 50.8 | 81.6 |
|  |  | Medical assistant | 1,481 | 545 | 36**.**8 | 31**.**4 | 42**.**3 | 232 | 42.5 | 37.8 | 47.6 | 300 | 55.1 | 50.0 | 60.1 |
|  |  | Nurse, midwife or HSA | 264 | 128 | 48**.**6 | 40**.**0 | 57**.**3 | 53 | 41.4 | 32.7 | 50.7 | 73 | 57.2 | 47.8 | 66.1 |
|  | Supervision | Supervision ever received | 1,593 | 609 | 38**.**3 | 33**.**8 | 42**.**9 | 240 | 39.3 | 34.7 | 44.1 | 354 | 58.1 | 53.2 | 62.8 |
|  |  | No supervision | 342 | 146 | 42**.**6 | 32**.**3 | 53**.**7 | 71 | 48.5 | 37.3 | 59.9 | 75 | 51.5 | 40.1 | 62.7 |
|  |  | Missing | 47 | 6 |  |  |  | 1 |  |  |  | 5 |  |  |  |
|  | RDT training | Training ever received | 1,291 | 513 | 39**.**8 | 35**.**0 | 44**.**7 | 198 | 38.6 | 34.1 | 43.3 | 307 | 59.9 | 55.2 | 64.3 |
|  |  | No training or updates | 644 | 242 | 37**.**6 | 28**.**9 | 47**.**1 | 113 | 46.5 | 36.9 | 56.3 | 128 | 50.3 | 40.4 | 60.2 |
|  |  | Missing | 47 | 6 |  |  |  | 1 |  |  |  | 5 |  |  |  |
|  | IMCI training | Training ever received | 939 | 367 | 39.1 | 33.4 | 45.0 | 145 | 39.6 | 34.3 | 45.2 | 215 | 58.7 | 53.2 | 64.0 |
|  |  | No training or updates | 996 | 388 | 39.0 | 32.6 | 45.8 | 165 | 42.5 | 35.9 | 49.4 | 214 | 55.0 | 47.8 | 62.0 |
|  |  | Missing | 47 | 6 |  |  |  | 1 |  |  |  | 5 |  |  |  |

Notes: Point estimates are weighted to account for unequal probabilities of selection due to differing client volumes on the interview date. Standard error estimation accounted for clustering of client observations within facilities. CI refers to confidence interval.
