# Supplementary material for: Integrated paediatric fever management and antibiotic over-treatment in Malawi health facilities: data mining a national facility census
Source: Malar J. 2016 Aug 4;15:396. doi: 10.1186/s12936-016-1439-7 (PMC4972956; doi:10.1186/s12936-016-1439-7)
Supplement: Supplementary file 2 — 10.1186/s12936-016-1439-7 Background characteristics of clients with fever complaints and antibiotic over-treatment, Malawi health facilities, 2013–2014. [file 12936_2016_1439_MOESM2_ESM.docx]

**Additional File 2: Background characteristics of clients with fever complaints and antibiotic over-treatment, Malawi health facilities, 2013-2014**

|  |  |  | **Without antibiotic need** | **Any antibiotic prescription (antibiotic over-treatment)** | | | |
| --- | --- | --- | --- | --- | --- | --- | --- |
|  |  |  | N | N | % | 95% CI | |
|  |  | **Total** | **1,411** | **830** | **58.8** | **55.1** | **62.4** |
| **Main** | RDT done prior to consultation | Yes | 541 | 282 | 52**.**2 | 46**.**6 | 57**.**7 |
|  |  | No | 870 | 548 | 62**.**9 | 57**.**8 | 67**.**8 |
|  | RDT result | Positive | 245 | 64 | 26**.**3 | 19**.**0 | 35**.**0 |
|  |  | Negative | 284 | 210 | 73**.**9 | 66**.**5 | 80**.**2 |
|  |  | Don't know | 12 | 8 | - | - | - |
|  |  | Missing | 871 | 548 | **-** | **-** | **-** |
| **Facility** | Type | Hospital (central, district, rural, other) | 498 | 316 | 63**.**5 | 55**.**4 | 71**.**0 |
|  |  | Health center/dispensary/clinic/post | 914 | 514 | 56**.**3 | 52**.**6 | 59**.**8 |
|  | Managing authority | Government | 1,076 | 629 | 58**.**5 | 53**.**8 | 62**.**9 |
|  |  | CHAM or other | 335 | 201 | 60**.**0 | 54**.**8 | 65**.**0 |
|  | Region | Northern | 231 | 140 | 60**.**7 | 49**.**2 | 71**.**1 |
|  |  | Central | 675 | 390 | 57**.**7 | 52**.**5 | 62**.**7 |
|  |  | Southern | 506 | 301 | 59**.**5 | 53**.**5 | 65**.**2 |
|  | Location | Urban | 438 | 276 | 63**.**0 | 54**.**4 | 70**.**9 |
|  |  | Rural | 973 | 554 | 56**.**9 | 53**.**1 | 60**.**7 |
|  | RDT stocks | RDT (at least one observed and valid) | 1,325 | 782 | 59**.**0 | 55**.**1 | 62**.**8 |
|  |  | RDT not seen, not valid or not available | 44 | 27 | 59**.**1 | 45**.**7 | 71**.**2 |
|  |  | Missing | 41 | 22 | - | - | - |
|  | Timer available | Facility or staff timer (at least one available) | 1,319 | 779 | 59**.**0 | 55**.**2 | 62**.**8 |
|  |  | Timer not available | 93 | 52 | 55**.**6 | 43**.**0 | 67**.**5 |
|  | Antibiotic stocks | Any antibiotic treatment (at least one observed and valid) | 1,408 | 829 | 58**.**9 | 55**.**1 | 62**.**5 |
|  |  | Any antibiotic treatment not seen, not valid, not available | 2 | 1 | 50**.**0 | - | - |
|  | Malaria risk | PfPR in 2-10 year olds [Mean, SD] | 1,411 | [15**.**7, 8**.**0] |  |  |  |
|  | Transmission season | Off-peak season | 1,205 | 719 | 59**.**7 | 55**.**6 | 63**.**7 |
|  |  | Peak season | 206 | 111 | 53**.**8 | 45**.**6 | 61**.**8 |
| **Patient** | Child's age | Age in months [Mean, SD] | 1,411 | [20**.**6, 15**.**1] |  |  |  |
|  | Maternal education | Primary school attendance | 978 | 569 | 58**.**2 | 54**.**2 | 62**.**1 |
|  |  | Secondary or more | 273 | 174 | 63**.**8 | 57**.**2 | 69**.**9 |
|  |  | Missing | 160 | 87 | - | - | - |
|  | CDB complaint | CDB complaint | 987 | 676 | 68.5 | 64**.**3 | 72**.**4 |
|  |  | No CDB | 424 | 154 | 36**.**4 | 30**.**9 | 42**.**1 |
|  | Diarrhea complaint | Diarrhea complaint | 618 | 1 | 51**.**4 | 45**.**4 | 57**.**4 |
|  |  | No diarrhea | 375 | 0 | 61**.**9 | 57**.**6 | 66**.**0 |
| **Provider** | Qualification | Doctor or clinical officer | 164 | 87 | 53**.**0 | 42**.**4 | 63**.**4 |
|  |  | Medical assistant | 1,055 | 630 | 59**.**7 | 55**.**5 | 63**.**7 |
|  |  | Nurse, midwife or health surveillance assistant | 192 | 114 | 59**.**1 | 51**.**0 | 66**.**7 |
|  | Supervision | Supervision ever received | 1,167 | 696 | 59**.**6 | 55**.**3 | 63**.**7 |
|  |  | No supervision | 225 | 123 | 54**.**5 | 44**.**8 | 63**.**9 |
|  |  | Missing | 19 | 12 | - | - | - |
|  | RDT training | Training ever received | 928 | 514 | 55**.**4 | 51**.**0 | 59**.**7 |
|  |  | No training or updates | 465 | 305 | 65**.**6 | 58**.**6 | 71**.**9 |
|  |  | Missing | 19 | 12 | - | - | - |
|  | IMCI training | Training ever received | 672 | 381 | 56**.**7 | 51**.**8 | 61**.**5 |
|  |  | No training or updates | 721 | 437 | 60**.**7 | 55**.**1 | 66**.**0 |
|  |  | Missing | 19 | 12 |  |  |  |
|  |  |  |  |  |  |  |  |
|  |  |  |  |  |  |  |  |

Notes: Point estimates are weighted to account for unequal probabilities of selection due to differing client volumes on the interview date. Standard error estimation accounted for clustering of client observations within facilities. CI refers to confidence interval.
